# Intestinal Transplant Immunology and Intestinal Graft Rejection: From Basic Mechanisms to Potential Biomarkers

**DOI:** 10.3390/ijms24054541

**Published:** 2023-02-25

**Authors:** Martin Rumbo, Mihai Oltean

**Affiliations:** 1 Instituto de Estudios Inmunológicos y Fisiopatológicos, Facultad de Ciencias Exactas, Universidad Nacional de La Plata—CONICET, Boulevard 120 y 62, La Plata 1900, Argentina; 2 The Transplant Institute, Sahlgrenska University Hospital, 413 45 Gothenburg, Sweden; 3 Department of Surgery at Institute of Clinical Sciences, Sahlgrenska Academy at the University of Gothenburg, 413 90 Gothenburg, Sweden

**Keywords:** intestinal transplantation, allorecognition, acute rejection, biomarkers, calprotectin, metabolomics, enterocytes, proteomics

## Abstract

Intestinal transplantation (ITx) remains a lifesaving option for patients suffering from irreversible intestinal failure and complications from total parenteral nutrition. Since its inception, it became obvious that intestinal grafts are highly immunogenic, due to their high lymphoid load, the abundance in epithelial cells and constant exposure to external antigens and microbiota. This combination of factors and several redundant effector pathways makes ITx immunobiology unique. To this complex immunologic situation, which leads to the highest rate of rejection among solid organs (>40%), there is added the lack of reliable non-invasive biomarkers, which would allow for frequent, convenient and reliable rejection surveillance. Numerous assays, of which several were previously used in inflammatory bowel disease, have been tested after ITx, but none have shown sufficient sensibility and/or specificity to be used alone for diagnosing acute rejection. Herein, we review and integrate the mechanistic aspects of graft rejection with the current knowledge of ITx immunobiology and summarize the quest for a noninvasive biomarker of rejection.

## 1. Introduction

Intestinal transplantation (ITx) remains a lifesaving option for patients suffering from irreversible intestinal failure and complications from total parenteral nutrition. Since the technique was established and became a clinical option in the late 1990s, it has been recognized that one of the outstanding features of this procedure was the strong allogenic response triggered by the graft, which has to be contained using more aggressive immunosuppressive regimes compared to other types of solid organ transplantations [1,2]. Considering the complexity of the tissue-resident immune cell populations along the gastrointestinal tract and the constant exposure to external antigens, including a diverse and wide microbiota, it was accepted that the intestinal graft is at the top of the list of high-immunogenic grafts. Pioneering work from K.A. Newell and coworkers in the end of the 1990s and early 2000s, elegantly performed in murine models, showed that acute cellular rejection is triggered by redundant effector pathways including CD4+ and CD8+ lymphocytes [3,4,5] and that blocking these effectors gives rise to chronic rejection, illustrating the redundancy of different immune-mediated effectors of rejection and the differences in costimulatory signals needed to trigger intestinal rejection by these populations [6,7,8,9]. In recent years, the use of immune activity surrogates such as the monitoring of DSA has been established in clinics and has allowed us to gain insight into the dynamics of alloresponse. Furthermore, we have progressed in our understanding of the immunology of the gastrointestinal tract and in aspects of solid organ alloresponse. The aim of this review is to integrate the present knowledge on ITx immunobiology with mechanistic aspects of graft rejection and review how this knowledge is bringing about new candidate biomarkers that may contribute to better monitoring of the graft status.

## 2. Basic Concepts of Intestinal Transplant Immunobiology

### 2.1. The Role of Direct Pathway, Semidirect Pathway and Indirect Pathway of Allorecognition in Intestinal Transplantation

Traditionally, it has been considered that there are several possibilities to elicit an allogenic immune response that will contribute to graft rejection [10]. The so-called direct pathway involves the presentation of allogenic molecules by antigen-presenting cells from the donor, which implies the presentation in the context of donor MHC molecules to the lymphocytes of the recipient. This pathway is mainly responsible for acute cellular rejection by the activation of CD4+ and CD8+ lymphocytes and, due to the limited lifespan of donor-derived antigen-presenting cells (APC), may not sustain alloresponse for longer than several months after transplantation. Depending on the number of antigen-presenting cells present in the graft, this pathway may constitute a serious threat to the survival of the allograft. In the case of intestinal transplantation, there are different professional APC populations either in the lamina propria or in organized lymphoid structures that may contribute to this mechanism [11,12,13]. Strong induction of immunosuppression, especially using depleting agents, aims to minimize this response. In addition, another important mechanism of alloresponse is the so-called indirect pathway, which involves the presentation of donor-derived allogenic peptides by recipient antigen-presenting cells using the exogenous antigen-presenting pathway [14]. Because of its nature, this pathway may be operative throughout the whole lifespan of a graft, as long as donor allogenic molecules (particularly donor MHC proteins and other alloantigens) are expressed in the graft. This pathway can mainly activate CD4+ lymphocytes by the presentation of allogenic molecules in the context of MHCII using the extrinsic pathway of antigen presentation. CD4+ lymphocytes activated by this pathway can provide cytokine signals to B cells specific to allogenic molecules, which is one important mechanism that contributes to the generation of donor-specific antibodies (DSA) [15]. The use of immunosuppressive agents that limit T cell activation is intended to limit the activation of this pathway.

Some years later, the semi-direct pathway was described [16,17,18], which implies the transference, mainly by extracellular vesicles, of functional donor-derived membrane-bound MHC molecules to recipient antigen-presenting cells, which afterward can present donor MHC molecules, potentially eliciting recipient CD8+ and CD4+ lymphocyte allogenic activation. This pathway can be operative indefinitely after transplantation and may be responsible for acute cellular rejection that may take place several years after transplantation, events that have been extensively documented in the ITx field [19,20,21].

### 2.2. The Role of Lymphoid Structures in the Alloresponse

**Figure 1:**
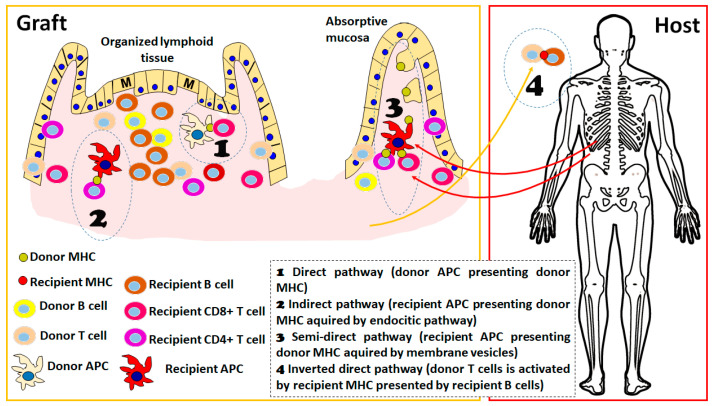
Schematic representation of different alloreactive activation mechanisms considered in the host vs. graft direction. The presence of organized lymphoid tissue in the graft is detailed since it favors the alloresponse; however, processes 1 to 3 could take place in both absorptive mucosa and organized lymphoid tissue. In the early post-transplant period, the 4 pathways are active, whereas in the long term, only processes 2 and 3 are sustained. The figure was produced using Microsoft PowerPoint^TM^.

Irrespective of the pathway that is eliciting the allogenic response, one feature that has been clearly established in experimental models of organ transplantation is the importance of the secondary lymphoid organs as sites of allorecognition and T cell activation. In the absence of secondary lymphoid organs in mouse models, the kinetics of alloresponse are much slower or absent, depending on the type of solid organ considered [22]. ITx has the particular feature of an important load of lymphoid structures in the graft, either the mesenteric lymph nodes included in the graft as part of the portal draining system or the multiple lymphoid structures present in the mucosa (Figure 1). There is important evidence that graft-organized lymphoid tissue is important for the development of rejection: in experimental models, it has been shown that recipient-derived lymphocytes are present in important amounts as early as 24 h after transplantation in mesenteric lymph nodes and Peyer patches of the graft [22] and that they expand and produce critical cytokines for acute rejection, such as interferon (IFN)-gamma, in the first days after transplantation. Furthermore, the absence of these structures in the graft completely abolishes the acute cellular rejection, indicating the importance of the direct pathway of recognition in this process.

Additionally, in human ITx, it has also been shown a fast turnover (more than 50% of the population at days 3 to 5 post-transplant) of the recipient-derived lymphocytes emerging through the mesenteric lymph [23] and also the presence of recipient-derived lymphocytes in the Peyer’s patches and isolated lymphoid follicles of the graft in the first 2 weeks after transplantation [24]. In recent years, due to different technical refinements [24,25], it was possible to appreciate the diversity of lymphoid structures present in the human mucosa, reinforcing the idea of the singularity of ITx from an immunological point of view. Although it is still incomplete, the understanding of the functional differences between structures in all cases of B cell compartments is present [25], and it has been shown that human ILF [24] and Peyer’s patches [26] contain TFH able to deliver T-B cooperation and that these structures are not affected by immunosuppression [24], raising the possibility that these sites are also important for the activation and expansion of donor-specific B cells, giving rise to donor-specific antibodies (DSA).

### 2.3. A New Pathway of Allogenic Immune Activation Operating in ITx

Recent evidence indicates the existence of a new activation pathway, the so-called inverted direct pathway, that is responsible for early de novo DSA formation. This pathway operates by the interaction of donor-derived CD4+ T lymphocytes with recipient B cells that may present in the context of recipient MHCII molecules and consequently act as direct alloactivators of donor T lymphocytes, operating in a way similar to the direct pathway with the particular feature that, in this case, donor vs. recipient cell roles are inverted [27]. From an immunological point of view, this is further evidence of the “two-ways allogenicity model” that we will describe below. In this case, the activation of donor-derived T cells is a feature of a graft vs. host response; however, the cytokines produced by the activated T cells contribute to the class switch and expansion of the host vs. graft B cell response (in this case, recipient B cells act as antigen-presenting cells of the recently described inverted direct pathway). Although this mechanism has been elegantly demonstrated in a mouse model and there are indications that it is acting on human solid organ transplantation, its relative importance still needs to be proven [28]. There is evidence that this could be an important source of DSA in clinical ITx since a correlation between the amount of passenger donor-derived T cells in the graft and the kinetics of the appearance of DSA in different types of transplants [27]. Furthermore, the presence of donor-derived B cells in the intestinal graft and intestinal graft-derived lymph has been described [23,29], indicating that this pathway could also be operating by the migration of recipient B cells into graft lymphoid structures and also by the emigration of donor-derived passenger T cells, as proposed by this recent study [27]. 

## 3. Emerging Concepts in Intestinal Transplant Immunobiology: The Two-Way Allorecognition Model and the Importance of Resident Memory T Cells

In the past few years, with the possibility of performing sequencing techniques that allow the identification of individual T cell clones and a clever combination of allostimulation experiments [30], pioneering work by Megan Sykes’ lab has shown that, upon solid organ transplantation, there is an expansion of effector T cells of donor origin (so-called graft versus host clones, GvH) concomitant with an expansion of effector T cells of recipient origin (host versus graft clones, HvG), in both cases triggered by the different alloactivation pathways described above. The magnitude of the GvH and HvG responses depends on the relative abundance of donor T cells in the graft [31,32,33]. Interestingly, the opposing activity of GvH and HvG responses may coexist under immunosuppressive regimes to a certain extent without consequences in the clinical aspect of the patient [31]. If one of these responses results in overexpansion, clinical rejection or graft versus host disease takes place. As expected, in the case of intestinal and multivisceral transplantation, the amount of T cells that are transferred with the graft makes the magnitude of the GvH response detectable, whereas it is less evident in other solid organ grafts [32]. In experimental rat models of multivisceral transplantation, this has also been evidenced by changing the lymphoid size of the graft upon inclusion of the spleen as part of the graft or removal of the recipient’s spleen [34]. The presence of lymphoid precursors in the intestinal graft has also been evidenced in a clinical setting by showing the repopulation of the recipient bone marrow with precursors of donor origin in the case of ITx [35,36], which is dependent on the initial expansion of GvH clones that, in part, due to their effector function, facilitate the population of the bone marrow niche by the donor-derived precursors. The recognition of the coexistence of these two opposite effector populations and their functional evolution may allow us to adjust immunosuppressive regimes or generate tolerogenic strategies based on improved knowledge of chimerism dynamics.

### Importance of Resident Memory T Cells

Resident memory T cells (Trm) are a subset of memory cells phenotypically and transcriptionally different from circulating effector and central memory T cells, which are mainly characterized by their non-circulating behavior, remaining long term, particularly in barrier organs such as skin, gut, lungs and reproductive tract, with the subset numerically the largest T cell memory subset. In recent years, it has been clearly established that in addition to its role in infectious immunity, this subset can be particularly relevant in the process of graft rejection [37]. Interestingly, using a mouse model of chronic kidney rejection based on the recognition of a transgenic OVA peptide ubiquitously expressed in tissues and recognized by transgenic TCR CD8 T cells, it was recently shown that resident memory CD8 T cells are central players in this model of chronic CD8-driven rejection [38]. Although authors transferred naïve OVA-specific CD8 T cells as drivers of rejection, over time it was observed that these allogenic cells activate in the kidney graft and acquire the phenotype of Trm CD8 cells. In spite of persistent antigenic exposure, Trms maintain activation markers without showing an exhaustion phenotype and are responsible for graft loss in this model. It is remarkable that, in spite of being a “non-frontier” organ, the kidney graft developed a strong proliferation of Trms at 4 weeks post-transplant, which do not recirculate, as shown through elegant parabiosis experiments. In another seminal study, a new mouse model of subsequent skin grafts was used to study the role of Trm in graft rejection: initially using an immunocompetent MHC mismatched recipient, allowing the generation of alloreactive CD4+ and CD8+ Trm that established in the skin, with a very low frequency of recirculation [39]. These animals were used as skin donors to be transplanted into SCID or IL2 gR-/- animals that are not able to mount T cell responses. In this second transplanted animal, the only source of alloreactive cells is the alloreactive Trm included in the graft. By using this animal as a recipient for the next skin transplant, authors could show that alloreactive Trm is sufficient to cause skin rejection. In this case, either CD8+ or CD4+ Trm are generated, as both populations are redundant in causing skin rejection. Interestingly, they also showed that, upon activation, there is a proliferation of Trm that, under these circumstances, can be detected in the circulation and spleen, indicating that part of the activity generated in the graft could be reflected in changes in peripheral blood, even in the case of Trm, allowing the possibility of monitoring the process. Intriguingly, the authors also performed heart transplantation in these recipient animals, which showed that skin alloreactive Trm is also able to reject heterotopic heart grafts, showing the capacity of alloreactive Trm to reject solid organs.

Authors have also transcriptionally and functionally characterized the profile of the alloreactive CD4+ Trm, which are mainly Th17-like cells in this model. In the field of clinical ITx, Kroemer et al. (2021) recently described Th17 cells as central players in keeping the progress of thymoglobulin-resistant graft rejection [40]. The characterization of these cells showed that they express a memory phenotype. Since the authors did not use antibody panels equivalent to those in previously mentioned studies [38,39], it is not possible to conclude exactly the role of Trm in this scenario, but this warrants future research on this topic. Recent work from Columbia University [31] showed that, in a clinical setting, there is a progressive replacement of intestinal graft donor lymphocytes by recipients, with higher turnover in the cases that develop clinical rejection, with remarkable expansion of specific HvG clones. Interestingly, this study indicated that these cell populations progressively acquire Trm phenotypes, reinforcing the concept that alloreactive Trms are mid- and long-term post-transplant central actors of the rejection process. The confirmation of this concept may have important implications since the capacity of different immunosuppressive drugs used for patient management have lower activity on memory cells compared with naïve cells.

Furthermore, considering the above-mentioned two-way alloreactivity pathway, it is important to consider that the intestinal graft harbors an important population of resident memory T cells [41,42] that may also contribute to the different possibilities of immunological activation, such as graft vs. host activity and also the inverted direct allorecognition pathway already mentioned. The different contributions of these different pathways to the clinical condition are yet to be established but may contribute to bringing additional options for patient management.

## 4. Open Questions on Immune Mechanisms Relevant to Rejection

Although the main drivers of alloresponse have been characterized, as described above, there are several mechanisms that have been described on homeostatic/physiopathogenic circuits in the intestinal mucosa that have not been explored in the context of intestinal transplantation.

MHC class II expression by intestinal epithelial cells (IECs) was described in the 1980s [43,44], and it has been shown that they may function as non-conventional APCs, interacting with T cells and shaping homeostatic circuits [45]. Their putative role as APCs in the different pathways of alloresponse has not been clearly established. It has been shown that IECs can secrete extracellular vesicles containing MHCII molecules capable of being acquired by mononuclear phagocytes: a circuit that participates in eliciting an adaptive response to microbial antigens [46]. These circuits can clearly participate in a semi-direct pathway of allogenic response in intestinal transplantation; however, their relevance in the rejection process has not been established yet. Furthermore, several unconventional lymphoid populations in the intestinal mucosa may participate in antigen-driven activation circuits [47]. Type 3 innate lymphocytes (ILC3) can also express MHCII and participate in antigen presentation to CD4+ conventional T cells [48], but rather than inducing T cell activation, they act as modulators of adaptive responses, inducing Tregs for commensal microbiota [49]. Deletion of MHCII in ILC3 induces dysregulation in CD+T cell responses that results in spontaneous intestinal inflammation [48]. Although ILC3 have been reported to have a protective role in intestinal rejection, possibly associated with their capacity to generate IL22 [50,51], their role as inducers of alloresponse has not been assessed. Still, in the same way, changes in the intraepithelial lymphoid compartment along intestinal transplantation follow-up have been described [52]; however, the contribution to alloresponse of several unconventional T cell populations, such as MAIT, CD8aa or gdT lymphocytes, is still not clearly established.

## 5. Biomarkers of ITx Rejection: An Imperfect Work in Progress

**Figure 2:**
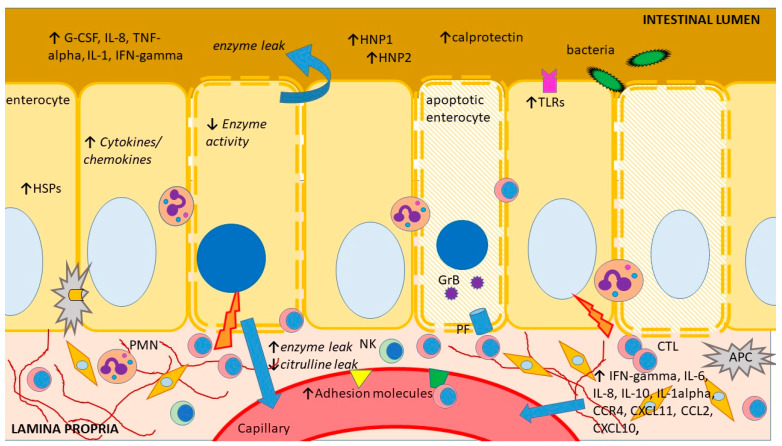
Schematic representation of the main sites and mechanisms of injury during intestinal acute rejection as well as an outline of its main consequences and features previously explored as biomarkers. APC—antigen-presenting cell, G-CSF—granulocyte colony-stimulating factor, CCR—CC chemokine receptors, CXCL—chemokine (C-X-C motif) ligand, CTL—cytotoxic T lymphocyte, GrB—granzyme B, HNP—human neutrophil protein, HSP—heat shock protein, IFN—interferon, IL—interleukin, NK—natural killer cell, PF perforin, PMN—polymorphonuclear, TLR—toll-like receptor, TNF—tumor necrosis factor. The figure was produced using Microsoft PowerPoint^TM^.

As of today, there are no noninvasive biomarkers showing adequate sensitivity and specificity to stand alone as diagnostic assays for intestinal acute cellular rejection (ACR). However, a number of biomolecules have been suggested as potential rejection markers, with some of them, such as plasma citrulline or fecal calprotectin, which are in clinical use. These biomolecules show altered expression during ACR, either due to tissue injury, the accompanying immune response activation and/or bystander inflammation or the ensuing metabolic changes in the rejecting intestinal graft (Figure 2). Unfortunately, tissue injury and immune response activation/inflammation also occur during other circumstances following ITx, such as ischemia-reperfusion injury or infectious enteritis, and the few biomolecules that are in clinical use so far can only be regarded as screening tools or exclusionary markers.

Below, we summarize the most significant findings and hypotheses explored so far according to three main pathophysiological mechanisms: direct graft injury secondary to the acute rejection process, local and systemic inflammation triggered by the alloimmune response and altered local metabolism following damage to the intestinal graft, explored via high throughput technologies. Many molecular events and subsequent cellular and molecular alterations are intertwined and dependent on the intensity and gravity of the immune response.

### 5.1. Markers of Graft Injury

Intestinal mucosa is the main target of the rejection process, and ultimately enterocyte loss ensues. Circulating levels of citrulline, a nonprotein amino acid produced mostly by enterocytes as the end product of glutamine metabolism, have been suggested as a measure of functional enterocyte mass. Citrulline is stable and relatively easy to analyze in plasma and dried blood spot samples [53]. In ITx recipients, serum citrulline levels have been reported to decrease during ACR and inversely correlate with its severity, prompting several investigators to suggest citrulline as a biomarker of acute rejection [54]. Unfortunately, a series of limitations related to the metabolism of citrulline, such as its variation related to body (and graft) size and renal function, have dampened the initial enthusiasm around it [55,56]. The individual variation generates a considerable overlap between plasma citrulline levels in patients with normal allograft histology and those with ACR. Additionally, the observation that citrulline concentrations in the early post-transplant period (up to three months) are decreased due to ischemia-reperfusion injury at a time when the incidence of ACR is potentially at its highest, further limits its usefulness. In spite of a rather satisfactory sensitivity (>80%), the specificity of low citrulline levels for rejection is modest (58%), and it cannot discern between rejection and other cases of citrulline decrease such as infectious enteritis [57]. Thus, the current belief is that citrulline levels reflect the extent of mucosal injury but do not seem to be a useful diagnostic marker for rejection or viral enteritis, as their values decline only when significant, widespread mucosal damage has occurred.

Several other biomolecules involved in enterocyte metabolism have been explored in the setting of intestinal ACR. Rodent studies have indicated that the intestinal fatty-acid binding protein (I-FABP), present primarily in mature, villus enterocytes which are normally undetectable in the serum, increased during ACR (196 ± 24 ng/mL), and rejection treatment with cyclosporine consistently reversed rejection and decreased I-FABP in rats [58]. However, a small clinical study of nine patients did not find meaningful elevations of serum and/or urine I-FABP in patients with histologically proven rejection [59]. Reasons behind the discordant results may have been represented by the low-lipid diets given early after transplantation, which may limit the expression of I-FABP [60], or the fact that only one out of four rejection episodes was severe and involved I-FABP-rich villus enterocytes, whereas the other three rejection episodes were mild/moderate and limited to the crypts.

The activity of histamine-degrading enzymes diamine oxidase and histamine N-methyltransferase (HNMT) was found to decrease in the mucosa of rejecting rat allografts and revealed a strong negative correlation with the histological rejection score, whereas isografts showed rather constant tissue enzyme activity up to eight days after ITx [61]. These two enzymes are relatively constant within each individual and bowel segment and reflect the functional integrity of the intestinal mucosa. Although serum and stool assays for the activity of these two mucosal enzymes are well established, no human studies have explored this hypothesis so far.

Enterocyte apoptosis is a central mechanism of tissue injury during intestinal ACR as one of the key effector mechanisms of cytotoxic T lymphocytes (CTL). Its increased occurrence is ubiquitous in different species, and its magnitude correlates with the severity of rejection [62,63,64]. Indeed, quantitation of apoptotic activity in the allograft (crypt cells) is one of the features used for the pathologic grading of the severity of rejection of the transplanted small intestine [64,65]. One of the death-inducing mechanisms used by CTLs is the exocytosis of granules containing perforin (a pore-forming protein) and several types of granzymes (serine proteases). Upon contact between CTLs and enterocytes, perforin facilitates the entry of granzymes into the target cell, where the latter can directly cleave the proapoptotic protein BH3 interacting-domain death agonist (BID) to its active form, which will then translocate to the mitochondria and increase its permeability. In the cytoplasm, granzymes cleave several substrates and induce cell death through the activation of caspase-dependent and -independent pathways. Both granzyme B and perforin were identified as co-expressed in the mucosa of rejecting intestinal allografts, correlating significantly with the histological severity of ACR [66]. A larger follow-up study from the same group analyzing granzyme B and perforin using total RNA extracted from peripheral blood mononuclear cells confirmed their increase during acute rejection and identified a sensitivity and specificity for acute rejection of 80% and 87% for granzyme B GB and 70% and 87% for perforin, respectively [67]. However, similar increases were found during post-transplant lymphoproliferative disease and viral enteritis, thus greatly reducing the diagnostic value of these two biomolecules. In addition, the study identified a “physiologic” increase in both molecules during the first four weeks after ITx and a high susceptibility of both these tests to antirejection treatment (steroid pulse or Thymoglobuline), both circumstances making the interpretation of the tests difficult in the case of acute rejection.

Increased serum levels of suppressor of tumorigenicity 2 (ST2), a regulatory decoy receptor of IL-33, have been observed during inflammatory bowel diseases [68]. In a small study of 18 pediatric intestinal transplant recipients with and without rejection or non-specific enteritis without rejection, ST2 levels were found to be significantly increased at the time of biopsy-proven rejection compared to rejection-free periods [69]. This increase appeared specific to rejection, as it differentiated rejection from non-specific enteritis. ROC analysis identified a cutoff of 3150 pg/mL and suggested a discriminative capacity for serum ST2 to distinguish rejection with a sensitivity of 62% and specificity of 72.2%.

A recent murine study using proteomics revealed significant alterations in the tissue expression of ninety-four proteins in the intestinal grafts that developed moderate rejection, compared to non-rejecting isografts [70]. Interestingly, the analysis of the canonic pathways revealed multiple known alterations corresponding to several metabolic pathways during intestinal rejection, such as citrulline biosynthesis, serotonin, dopamine or arginine degradation. The analysis also evidenced previously unknown changes in the tissue expression of numerous structural proteins, enzymes and components of several cellular signaling pathways, which could be further explored up- or downstream in search of potential biomolecules or metabolites useful as rejection biomarkers. One such biomolecule could be chromogranin A, which was found to decrease in the rejecting intestines in the confirmatory analysis of the study.

### 5.2. Markers of Local and Systemic Immunologic and Inflammatory Activation

The initial inflammation in the early stages of allorecognition initiates multiple phenotypic changes in the graft. Earlier experimental studies have indicated numerous alterations, including progressive mucosal pro-inflammatory activation [71,72], heat stress response [73] or graft dysmotility [74], occurring during intestinal acute rejection. Although many of these changes have been indicated, mostly evidenced in rodent transplant models without the use of immunosuppression, their confirmation in a clinical setting is still incomplete, and results based on human biopsies are mandatory for any meaningful advancement in the search for biomarkers.

Cytokines and their receptors play key roles in the activation and propagation of the alloimmune response through their chemotactic effects, inducing the expression of adhesion molecules, promoting the proliferation and differentiation of specific alloreactive T and B cell clones or having a direct cytotoxic effect on the allograft cells [75,76]. Several experimental studies [71,72,77,78,79,80] reported a significant and sustained increase in several cytokines and adhesion molecules in rejecting intestinal allografts. However, essentially all these studies focused on the intragraft expression of these molecules, and the information on this topic in body fluids remains surprisingly limited.

In spite of the early enthusiasm around the potential of various cytokines to diagnose intestinal ACR, there seems to be a gap between the experimental and clinical data, and only a few clinical reports have been published and are based on a small number of analytes and patients [81,82,83,84]. These limited, early observations showed increased soluble adhesion molecules or cytokines in patients rejecting their intestinal allografts but generally failed to identify clear patterns, thresholds or roles for them. A more recent study performed on formalin-fixed, paraffin-embedded, human mucosal biopsies investigated the expression of 280 genes involved in immune response, inflammation and apoptosis and found 92 genes showing significantly different expression levels between rejecting and non-rejecting intestines [85]. These genes included several cytokines and chemokines, endothelial adhesion molecules (ICAM-1, E-selectin), numerous epitopes involved in allorecognition (likely reflecting the immune cells’ infiltration), as well as multiple receptors for bradykinins, leukotrienes or cytokines. As all the samples selected for this analysis had moderate or severe ACR, it is still unclear which changes are associated with the earlier stages of rejection, which may be used for diagnostic or early detection as part of a surveillance strategy. In addition, no graft samples presenting confounding pathology such as infectious enteritis were analyzed, leaving the specificity of these findings unclear. Another shortcoming of this study is the continuous need for graft biopsies, whereas a non-invasive biomarker would ideally be identified in the stool or in the blood. A reason for the paucity of clinical studies may be the perceived confounding effect of immunosuppression (tolerance induction regimens, maintenance immunosuppression) and its impact on the kinetics of various biomolecules [86,87], adding to all the other causes of heterogeneity encountered in a clinical setting. Indeed, a clinical study found that, whereas the expression of intragraft IFN-gamma, CXCL10, and CXCL11 was clearly increased during rejection, rejecting individuals receiving reduced immunosuppression showed a 13-fold increase in IFN-gamma expression and a 9-fold increase in CXCL10 expression, while patients with more intense immunosuppression revealed a significantly lower increase [88].

A small pilot study found an increase in plasma regenerating islet-derived 3-alpha (REG 3-alpha), a C-type lectin antimicrobial peptide synthesized by enterocytes and Paneth cells, during intestinal acute rejection [89]. As the REG3-alpha increase seems to have also heralded rejection within 1 week, it is possible that this molecule will attract more attention in an effort to determine its value in terms of specificity and sensitivity in the setting of intestinal transplantation.

A relatively recent and still evolving paradigm in the follow-up of ITx patients is the monitoring of the development of humoral immunity against the graft. De novo production of antibodies against the graft’s human leukocyte antigens (HLA), the so-called donor-specific HLA antibodies (DSA), has been linked with a higher risk of ACR in several types of organ transplants, including ITx [90,91,92,93,94]. A detailed account of the type, impact and management of DSAs after ITx is beyond the scope of this review. Nonetheless, it is worth mentioning that several independent studies analyzed the relationship between de novo DSAs and outcomes, including ACR, and most could not identify a clear, significant relationship between de novo DSA and ACR. However, most studies suggest that long-term outcomes were poorer in patients developing de novo DSAs, and DSAs can be considered an indicator of ongoing, low-grade immunologic activity against the intestinal allograft.

A different and more pragmatic approach has been represented in the analysis of the ostomy effluent. Whereas this approach is less used in rodents, it allows easy, non-invasive access to abundant biological material originating in or having direct contact with the intestinal mucosa, the same site where rejection occurs. Fecal calprotectin levels (FCL) have been routinely used in practice over the last few decades to initially differentiate between inflammatory bowel disease (IBD) and irritable bowel syndrome (IBS), as well as to assess mucosal healing or the recurrence of inflammation in the follow-up of IBD patients. Calprotectin is a calcium- and zinc-binding protein of the S-100 protein family, mainly found within neutrophils, and its presence in feces is due to neutrophil infiltration into the gastrointestinal tissue secondary to an infection or an inflammatory process. Fecal calprotectin is homogenously distributed in stools and is very resistant to in vivo degradation (pancreatic secretions and intestinal proteases, bacterial degradation) and in vitro degradation (several days at room temperature). The first article on the analysis of calprotectin in the stomal fluid of intestinal transplant recipients reported increased FCL both during ACR rejection and non-specific enteritis and found an optimal cut-off level for calprotectin in predicting the presence or absence of intestinal allograft rejection at 92 mg/L with 77% specificity and 83% sensitivity [95]. This interesting report was soon followed by several other independent analyses that confirmed FCL is consistently increased during rejection compared with normal patients, but it has a rather low specificity for intestinal rejection [96,97,98]. In spite of the common conclusion that FCL alone cannot satisfactorily discriminate between rejection and inflammation or infection, there is a general perception that FCL could be used as a first-line test for continuous evaluation of intestinal graft status, as it may be useful to exclude inflammatory pathology and thus reduce the need for graft endoscopy by up to 45%.

A combined approach using a multiplexed analysis of 17 cytokines and high-throughput proteomics of the ostomal effluent of 16 intestinal transplant recipients found increased levels of five innate immune cytokines early post-transplantation (granulocyte colony-stimulating factor, interleukin (IL)-8, tumor necrosis factor (TNF)-alpha, IL-1beta and IFNγ), but only IFNγ levels were significantly higher in samples with rejection [99]. Proteomic analysis revealed 17 proteins differentially seen in rejection, with three of them identified as human neutrophil peptides 1, 2 and 5. These proteins, which belong to the alpha-defensins (antimicrobial peptides), were also found to elevate early in the post-transplant stage and could reflect the rejection-associated innate immunity. Although the significance of these findings is unclear, this analysis suggests the potential presence of more interesting biomolecules in the stomal effluent, which may deserve further analysis.

### 5.3. The Microbiome after ITx

The presence of a very large, complex and dynamic microbial microenvironment makes the intestinal graft unique. The recent advances in sequencing technology have allowed the high-resolution analysis of the intestinal microbiome, unraveled the profound effects of intestinal microbiota on their hosts and involved the occurrence of multiple, highly diverse diseases, such as asthma [100], diabetes [101] and colon cancer [102], to name only a few. The high incidence of infections following ITx [103,104] mandates prolonged and repeated use of antibiotics, which alter the intestinal microbiome. Several immunosuppressive drugs currently used also have antibiotic activity [105,106], and changes in microbiome profiles during different immunosuppression regimens have been reported, suggesting that immunosuppression has an impact on the human microbial population [107]. In addition, the presence of feeding tubes and ostomies alters the normal microbial ecology of the transplanted intestine [108]. Thus, a shift in the microbiome from an aerobic–anaerobic condition to a predominantly anaerobic condition has been found following ileostomy removal and the restoration of intestinal continuity in intestinal transplant recipients, although the small bowel did not seem to be negatively affected by this.

In a clinical study on 19 ITx recipients, comparisons between samples from non-rejecting ITx and ITx with ACR revealed major alterations in the proportions of multiple bacterial taxa associated with active rejection [109]. At the phylum level, it has been reported that there was a reduction in Firmicutes (from 81 to 29%) and an expansion of Proteobacteria (from 16 to 61%) during manifest ACR, whereas at the family level, a decrease in Streptococcaceae, Enterococcaceae and Lactobacillaceae was observed. However, the study did not detect clear, significant differences between non-rejecting ITx and ITx in the period preceding ACR, which could be used as early biomarkers, although the ITx microbiota in the pre-rejection period showed the same trend as that found during ACR. Part of these findings was confirmed by a recent analysis of 43 ITx recipients, where patients without ACR beyond the first 6 months after transplantation and normal ITx function showed a large predominance of Firmicutes and a composition of other phyla, including Proteobacteria and Bacteroidetes, that was closer to the general population. In contrast, the transplanted microbiome showed an Enterococcus-dominant dysbiosis and a relative increase in Enterococcous and Fuminococcus [110].

Although a direct cause–effect relationship between the microbiome alterations (including the enterococcus predominance) and the ACR and its role in graft monitoring remains to be established, these initial findings have important potential clinical implications in terms of patient management, as future targeted strategies to control the degree of intestinal Enterococcus colonization could have a beneficial impact on graft and patient outcome.

### 5.4. The Omics Approach in the Search for Biomarkers

The last four decades of experimental and clinical research have identified hundreds of molecules that may be altered during the intestinal ACR, with some having the potential to assist with its diagnosis, prognosis and follow-up. Unfortunately, very few, if any, have shown true value in a clinical setting. This may be due both to the differences between experimental and clinical settings, the small size and inherent heterogeneity of clinical studies, as well as the technical and biological limitations of the approaches used. Nowadays, it is believed that either an entirely new, intestine-specific assay or a panel of less specific tests would be necessary to increase the accuracy of non-invasive rejection monitoring. The application of new analytical and bioinformatics techniques would allow a comprehensive analysis of complex biological samples and the simultaneous detection of a large number of chemically diverse analytes. This has already been applied in kidney transplantation, where a composite metabolite–mRNA signature in urine was diagnostic of ACR with high accuracy [111,112]. In ITx, the recent technical developments and progress of high-throughput omics technologies have already identified some additional molecules or biological pathways with potential biomarker value, which are summarized below.

In 36 ITx patients receiving different types of visceral allografts, metabolomics of the stomal effluent or feces demonstrated different metabolomic profiles between rejection and nonrejection [113]. A total of 477 (19%) of the 2541 detected metabolites revealed a significant fold change between rejection and nonrejection, and, following the examination of several databases, the metabolites with the most significant fold change between rejection and nonrejection were identified as leukotriene E4 (a metabolite of arachidonic acid), D-pantethine, the dimeric form of pantothenic acid (vitamin B5), pyridoxal-5-phosphate (vitamin B6), taurocholate (a bile salt of taurocholic acid) and riboflavin (vitamin B2). Whether these analytes themselves could be useful as new biomarkers of intestinal rejection or other components of their metabolic pathways are affected by rejection and could be analyzed remains unclear.

A second high-throughput analysis using quantitative proteomics with iTRAQ-labeling and mass spectrometry performed in rejecting murine intestines and mentioned earlier [70] identified 86 proteins differentially expressed in rejecting allografts versus non-rejecting isografts (variation > 20%) and an alteration pattern unique to the rejecting allografts: thirty-seven proteins and enzymes (including S100-A8 and IDO-1) were significantly upregulated, whereas forty-nine (among other chromogranins, ornithine aminotransferase and arginase) were downregulated. Following the exclusion of eight proteins that revealed the same alteration pattern as that found in syngeneic grafts and were likely unrelated to AR, 86 proteins continued to reveal an alteration pattern only found in the rejecting allografts. These changes involved multiple metabolic pathways, whose secondary metabolites and downstream metabolic processes may reveal potential biomarkers for intestinal AR.

## 6. Conclusions and Future Directions

The intestinal graft appears to have a unique immunology due to its contact with external antigens and the exposure to the intestinal microbiota, the gut-associated lymphoid tissue following with the graft, as well as the abundance of epithelial cells. Graft rejection occurs more frequently than in any other type of organ transplant, in spite of potent immunosuppression, and may rapidly result in the loss of the mucosal barrier, life-threatening sepsis and patient death. At the same time, the transplanted intestine lacks reliable, specific, noninvasive biomarkers, allowing non-invasive rejection surveillance. As most of the non-invasive assays tested to date in ITx are the same as those used in the management of inflammatory bowel disease, they all lack specificity by default.

Future research should continue the quest to improve our understanding of two-way allogenicity, with an emphasis on the generation of regulatory populations that may reduce the burden of immunosuppression. Additionally, further research efforts should continue to explore the development of assays capable of determining the involvement of memory T cells in the graft rejection process and their potential translational use. Likewise, advances in the monitoring of other types of allografts, such as circulating cell-free donor (i.e., graft) DNA or the alterations in the microbiota that may herald or favor ACR, should be tested in adequately sized, multicenter studies on either prospectively collected material or on existing biobank samples. The continuous quest for the identification of well-defined alterations occurring during rejection in the main targets of the immune response, that is, the intestinal mucosa and its microenvironment, could ultimately reveal the long-sought and badly needed rejection biomarker.

## Data Availability

Not applicable.

## Abbreviations

ACR	acute cellular rejection
APC	antigen-presenting cell,
G-CSF	granulocyte colony stimulating factor,
CCR	CC chemokine receptors,
CXCL	chemokine (C-X-C motif) ligand
CD	cluster of differentiation
CTL	cytotoxic T lymphocyte
DSA	donor specific antibody
FCL	fecal calprotectin levels
GvH	graft versus host
HLA	human leukocyte antigen
HNMT	histamine N-methyltransferase
HNP	human neutrophil protein
HvG	host versus graft
IBD	inflammatory bowel disease
IBS	irritable bowel syndrome
IEC	intestinal epithelial cell
IFN	interferon
IL	interleukin
ILC3	Type 3 Innate lymphocyte
ILF	isolated lymphoid follicles
ITx	intestinal transplantation
MAIT	mucosal associated invariant T cell
MHC	major histocompatibility complex
REG 3-alpha	regenerating islet-derived 3-alpha
SCID	severe combined immunodefficient
ST2	Suppressor of Tumorigenicity 2
Th	T-helper cell
TNF	tumor necrosis factor.
Trm	resident memory T cells
